# In Vitro and In Vivo Evaluation of an Emollient‐Rich Moisturizer Developed to Address Three Critical Elements of Natural Moisturization

**DOI:** 10.1111/jocd.70085

**Published:** 2025-04-03

**Authors:** Zoe D. Draelos, Diane B. Nelson

**Affiliations:** ^1^ Dermatology Consulting Services PLLC High Point North Carolina USA; ^2^ skinbetter science, a Dermatological Beauty Brand of L'Oréal USA Inc. Phoenix Arizona USA

## Abstract

**Background:**

A new, emollient‐rich moisturizing cream has been developed to support three critical elements of natural skin moisturization—hyaluronic acid, natural moisturizing factors, and lipids.

**Aims:**

The aim of this study was to evaluate in vitro biomarkers associated with skin hydration and barrier support, followed by in vivo clinical hydration assessment and tolerability.

**Methods:**

Using an in vitro epidermal skin model, tissues were treated with the study moisturizer or control (saline) for 24 h. Genes associated with hydration and barrier support were analyzed: claudin 4 (CLD4), aquaporin 3 (AQP3), hyaluronic acid synthase 2 (HAS2), and hyaluronidase 1 (HYAL1). The clinical study evaluated twice‐daily use of the study moisturizer in subjects with moderate‐to‐severe dry skin. Subject satisfaction and skin hydration measurements were captured at baseline, 2, 4, and 8 weeks.

**Results:**

Increased expression of CLD4, AQP3, and HAS2 and reduced activity of HYAL1 were demonstrated after 24 h. In subjects applying the study moisturizer, significant mean percent improvements from baseline in skin hydration occurred at Weeks 2 (41%; *p* < 0.0001), 4 (38%; *p* < 0.0001), and 8 (116%; *p* < 0.0001). Ninety‐six percent of subjects reported their skin felt hydrated after 8 weeks.

**Conclusions:**

An emollient‐rich moisturizing cream developed to support three critical elements of natural skin moisturization increased the expression of biomarkers associated with skin barrier support and hydration, and reduced the expression of HA‐degrading enzymes. Early, significant increases in skin hydration were observed.

## 1 Introduction

Youthful skin is characterized by resilience, pliability, and turgor. In large part, this is due to its elevated water content, which contributes to a strong and healthy skin barrier. The natural aging process, along with cumulative exposure to environmental insults, leads to a significant reduction in the skin's ability to retain water, resulting in a weaker skin barrier that is more vulnerable to insult, impaired wound healing, increased inflammation and infection, xerosis, and accelerated skin aging [1, 2, 3, 4, 5, 6, 7, 8, 9]. While hydrated skin constantly renews itself to maintain the protective functions of the skin barrier [3], dehydrated skin compromises the skin's barrier [4, 10]. Thus, it is critical for the skin to have sufficient hydration to maintain the integrity of the skin barrier, facilitate optimal functioning [3], and retain a more youthful appearance. In addition to the consistent use of photoprotection, aging, depleted skin requires continuous replenishment of natural components that support skin moisturization to maintain the resilience, pliability, and turgor associated with a youthful, well‐moisturized skin barrier.

## 2 Three Critical Components of Natural Skin Moisturization

### 2.1 Hyaluronic Acid

Most abundant in skin and a predominant component of the cutaneous extracellular matrix (ECM), hyaluronan or hyaluronic acid (HA), is a glycosaminoglycan (GAG). Composed of linear, repeating, polymeric disaccharide units and found in tissues and fluids throughout the body, HA helps to stabilize, and is stabilized by, other structural components of the ECM [11]. The flexible nature of the HA units, along with their hydrophilic properties, contributes to their ability to reinforce extracellular spaces within the dermis. HA is deposited as a high molecular weight polymer possessing anti‐inflammatory and antiangiogenic properties and is degraded to low molecular weight fragments and oligosaccharides, which have been purported to have proinflammatory, proangiogenic, and oncogenic effects [12].

HA also has a unique capacity to bind 1000‐fold of its weight in water and retain water molecules in the dermis and epidermis, critical to maintaining structure and volume. HA has a high turnover rate in skin, with a substantially shorter half‐life in the epidermis (2–3 h) than in the dermis (< 1 day) [11]. The natural process of aging leads to a marked reduction of HA in the epidermal layers and thus a loss of skin moisture [13]. Topical application of HA has been shown to improve skin hydration and rejuvenation [14]. Optimal hydration occurs when HA is maximized and hyaluronidase (HYAL), the enzyme that degrades HA, is minimized.

### 2.2 Natural Moisturizing Factors

Another vital component of natural skin moisturization contributing to optimal skin hydration involves a complex mixture of compounds referred to as natural moisturizing factors (NMF) [15]. NMF are highly efficient humectants comprised of amino acids and their derivatives, including pyrrolidone carboxylic acid (PCA), lactic acid, and urea; these are derived from the proteolysis of the protein filaggrin and its precursor profilaggrin [16]. Reductions in NMF can also occur with routine bathing and ultraviolet (UV) exposure. As with HA, aging has been shown to substantially diminish the concentration of amino acids in the stratum corneum, resulting in reduced hydration [16]. Specifically, research has shown that the water content of the stratum corneum gradually decreases with age, with variable effects on specific amino acids [15].

### 2.3 The Lipid Bilayer

In addition to maintaining hydration and humectancy, optimal function of the stratum corneum requires the formation and maintenance of an intercellular lipid membrane with a specific composition and lamellar structure [17]. As such, the third essential component in supporting a healthy skin barrier is achieving optimal levels of cholesterol, essential fatty acids, and sphingolipids or ceramides. This lipid layer comprises about 20% of the volume of the stratum corneum, of which 40%–50% are ceramides, 25% are cholesterols, and 10%–15% are free fatty acids [17].

Early studies indicated that the application of optimized, cholesterol‐dominant lipid mixtures facilitated accelerated stratum corneum barrier recovery in aged skin, whereas the application of occlusive agents failed to permeate the stratum corneum to facilitate recovery [18]. More recently, studies on ceramide‐dominant moisturizers have been found to specifically facilitate barrier repair in patients with skin disorders such as atopic dermatitis or psoriasis [17].

## 3 Evaluation of a New, Emollient‐Rich Moisturizing Cream

Moisturizers are an essential component of a skincare regimen [17, 19], ideally supplementing the skin's natural moisturizing systems, supporting barrier function, and facilitating hydration [20]. Active ingredients may include emollients to soften the skin, occlusives to form a barrier and prevent transepidermal water loss (TEWL), and humectants to bind and retain water in the stratum corneum [16, 17, 21].

The study moisturizer (TRMT‐L) supplements skin with HA, NMF, and lipids to support skin hydration and strengthen the skin barrier. Additional ingredients to enrich this formulation include glycine, niacinamide, and *Olea europaea* (olive) fruit extract, an antioxidant with anti‐inflammatory and humectant properties that helps hydrate and soothes skin (Table 1). Herein, we describe in vitro gene expression analysis of biomarkers associated with hydration and support of the skin barrier, as well as clinical evaluation of twice‐daily application of the study moisturizer over 8 weeks.

**TABLE 1:** Key ingredients.

Key ingredients	
Natural moisturizing factors	Urea complex
	Sodium PCA
	*Salicornia herbacea* extract
	Carnosine
	Sodium lactate
Barrier function	Cholesterol
	Ceramides 1 and 3
	Linoleic and linolenic acids
	Squalane
Hyaluronic acid	Aminopropyl ascorbyl phosphate
	Sodium lactate
	Sodium hyaluronate
Support	Glycerin
	Caffeine
	Jojoba esters
Trio‐L	Glycine
	Niacinamide
	*Olea europaea* (olive) fruit extract

## 4 In Vitro Gene Expression Analysis

The purpose of this in vitro study was to evaluate the expression of key biomarkers associated with skin barrier support and hydration utilizing an in vitro epidermal skin model. Selected biomarkers included the evaluation of aquaporin 3 (AQP3), claudin 4 (CLD4), hyaluronic acid synthase (HAS), and hyaluronan (HYAL). Aquaporin is a transmembrane protein that facilitates the transportation of water, urea, and glycerol within the epidermis and plays a role in cell migration, skin hydration, and biosynthesis [22]. AQP3 is the most predominant type of AQP found in the epidermis [22]. Claudins are barrier‐forming proteins and are part of a complex network of tight junction proteins involved in the regulation (barrier permeability) and selective diffusion of solutes throughout the epidermis and stratum corneum [23]. CLD4 is expressed in epithelial tissues throughout the body, including the epidermis. The expression of hyaluronic acid synthase (HAS), the enzymes responsible for hyaluronan synthesis and support, and HYAL, the enzymes that degrade HA, were also evaluated.

### 4.1 Methods

This study utilized an in vitro reconstructed epidermis comprised of normal human‐derived epidermal keratinocytes in a stratified corneal layer, and a dermal component containing an intact dermal–epidermal junction and viable dermal fibroblasts (MatTek, Ashland, MA). Tissues were equilibrated overnight at 37°C with 5% CO_2_ and ~95% relative humidity. The following day, equilibration medium was removed from each well and replaced with 2.5 mL of fresh maintenance medium. Each group (*N* = 4) was treated with 15 μL of either the study moisturizer or control (0.9% saline) applied to the center of each culture. The cultures were then returned to the incubator at 37°C with 5% CO_2_ and 95% relative humidity for 10 h (morning application). After 10 h, the products were rinsed, and excess liquid was removed with a sterile swab, and the products were reapplied. The tissues were returned to the incubator for 14 h (evening application). Twenty‐four hours after the initial application, tissues were rinsed and collected for quantitative polymerase chain reaction (qPCR) analysis. Each gene was assayed in duplicate. Gene expression was analyzed for AQP3, CLD4, HAS2, and HYAL1. Statistical analysis involved unpaired t‐tests (*p* < 0.05) relative to control; fold change (FC) values ≥ 2.0 were considered biologically relevant.

To ensure the formulation being tested was nontoxic to the skin model, cytotoxicity was assessed using media collected from the culture wells 24 h after the initial treatment, using a lactate dehydrogenase (LDH) cytotoxicity detection kit. Increased LDH activity is an indicator of damaged or dead cells.

### 4.2 Results

Cytotoxicity analysis on the formulation tested demonstrated low levels of cytotoxicity throughout the study.

**FIGURE 1:**
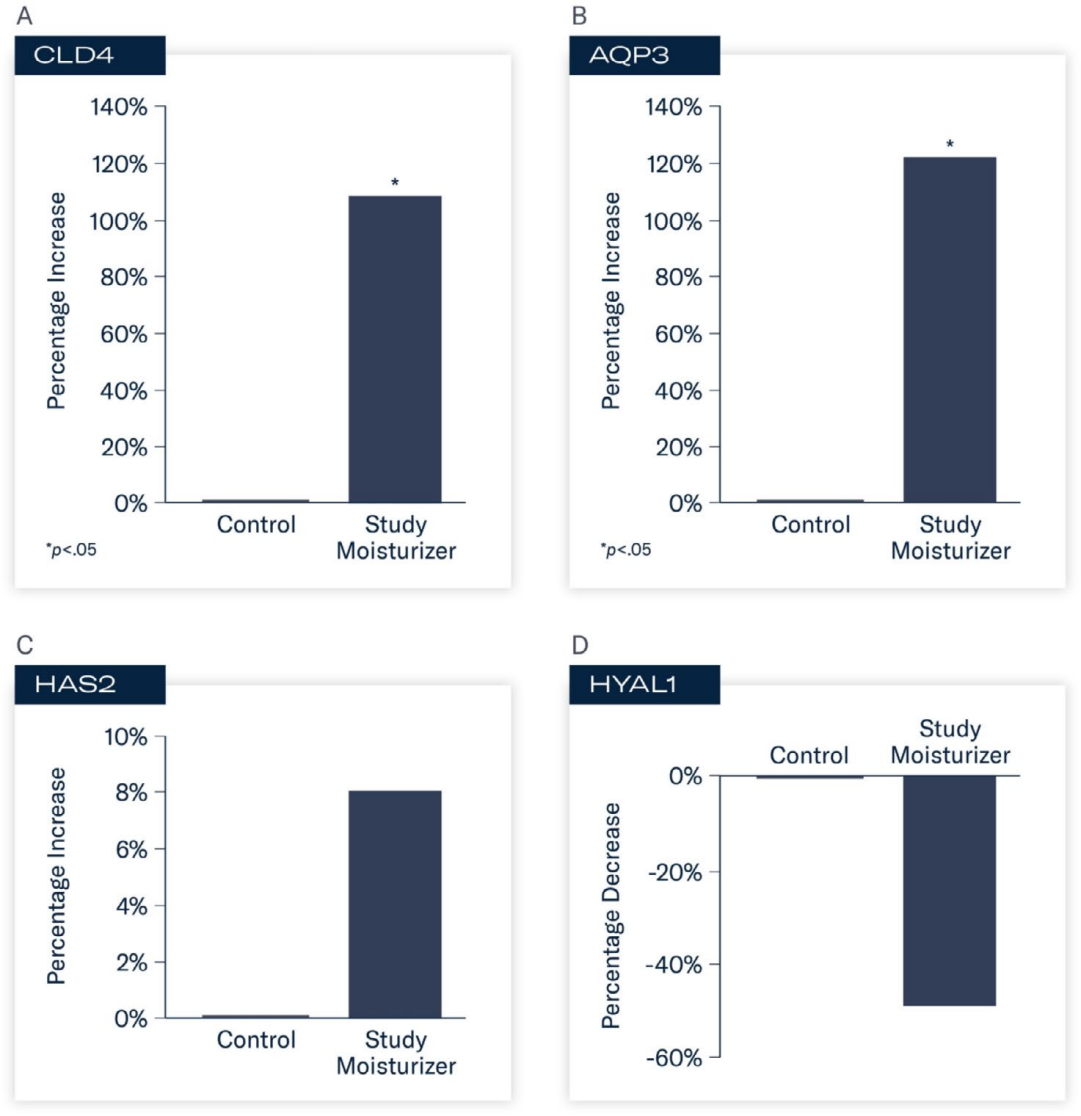
A–D Mean percent change in expression of biomarkers associated with skin barrier support and hydration.

At 24 h, statistically significant changes in gene expression were observed in tissues treated with the study moisturizer compared to control. Increased activity of CLD4 (FC 2.07, 107%; *p* < 0.05) and AQP3 (FC 2.21, 121%; *p* < 0.05) occurred. Enhanced activity of HAS2 (FC 1.08, 8%) was demonstrated with decreased activity of HYAL1 (FC –1.96, −49%). These results indicate enhanced support of proteins that play critical roles in skin barrier function and hydration, and protection against enzymes that degrade hyaluronic acid (Figure 1A–D).

## 5 In Vivo Evaluation of Moisturizing Effects and Subject Satisfaction

### 5.1 Methods

This single‐center, open‐label clinical trial enrolled subjects aged 50–70 years with Fitzpatrick skin types (FST) I–VI and moderate‐to‐severe dry, dehydrated facial skin in January and February 2023.

Subjects were deemed eligible for inclusion in the study following a 3‐day washout of any moisturizer; a 2‐week washout of cosmetic products containing alpha hydroxy acids (AHAs), beta hydroxy acids (BHAs), antioxidants, peptides, growth factors, nonprescription retinoids, or like products; a 4‐week washout of prescription‐strength hydroquinone or a topical retinoid; and a 3‐month washout of topical prescription corticosteroids or medications for rosacea. Exclusion criteria included severe presentation of lines or wrinkles, laxity, dyschromia, and erythema; dermatological disorders (e.g., acne vulgaris, psoriasis, and atopic dermatitis), melasma, or postinflammatory hyperpigmentation; severe rosacea; facial scarring; and subjects who were pregnant, lactating, or planning a pregnancy during the study period. All subjects provided written, informed consent prior to study participation.

Facial digital images were obtained at baseline, 2, 4, and 8 weeks using VISIA‐CR digital imaging (Canfield Scientific Inc., Parsippany, NJ). Assessment of skin hydration was measured utilizing noninvasive bioinstrumentation (Corneometer, Cortex Technology, Hadsund, Denmark) at baseline, 2, 4, and 8 weeks. Three skin surface hydration measurements were obtained, and an average reading was recorded from a predetermined location on the central right cheek. Subjects were instructed not to use the study product or sunscreen the morning prior to their study visit so as not to interfere with instrumentation measurements. At each study visit, subjects completed a self‐assessment questionnaire capturing their perception of the appearance of their facial skin, the effect of the study product on their facial skin, and product attributes, including 10 statements assessing their initial impressions of the study product and its effects approximately 30 min following their first application (in‐clinic, baseline visit). Adverse events (AEs) were obtained throughout the study period.

In addition to twice‐daily facial application of the study product, subjects were provided with a standardized skincare regimen, including a gentle cleanser and a sheer‐broad‐spectrum mineral‐based sunscreen stick (SPF 56 sunscreen).

### 5.2 Results

#### 5.2.1 Demographics

Twenty‐seven subjects were enrolled and completed the study. Subjects were female with a mean age of 62 years. Seventy‐four percent of subjects were FST I–II, 7% were FST III, and 19% were FST V–VI. Seventy‐eight percent (78%) of subjects were Caucasian/Whit, and 18% were African American/Black. Four percent (4%) of enrolled subjects were Hispanic.

#### 5.2.2 Skin Hydration Evaluation

**FIGURE 2:**
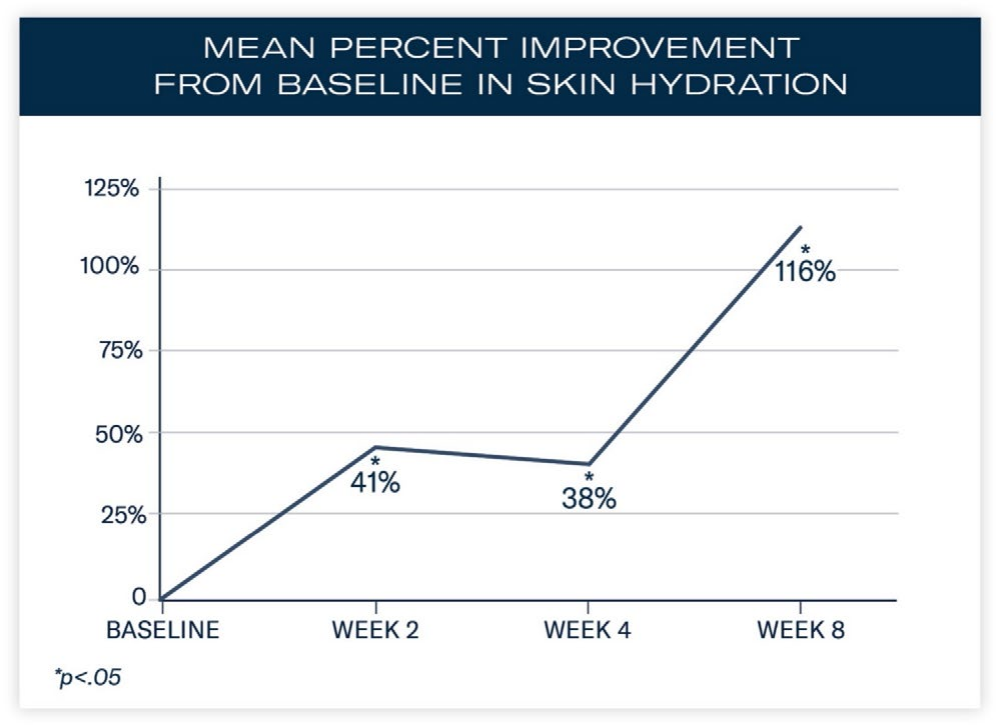
Quantification of skin hydration over 8 weeks.

**FIGURE 3:**
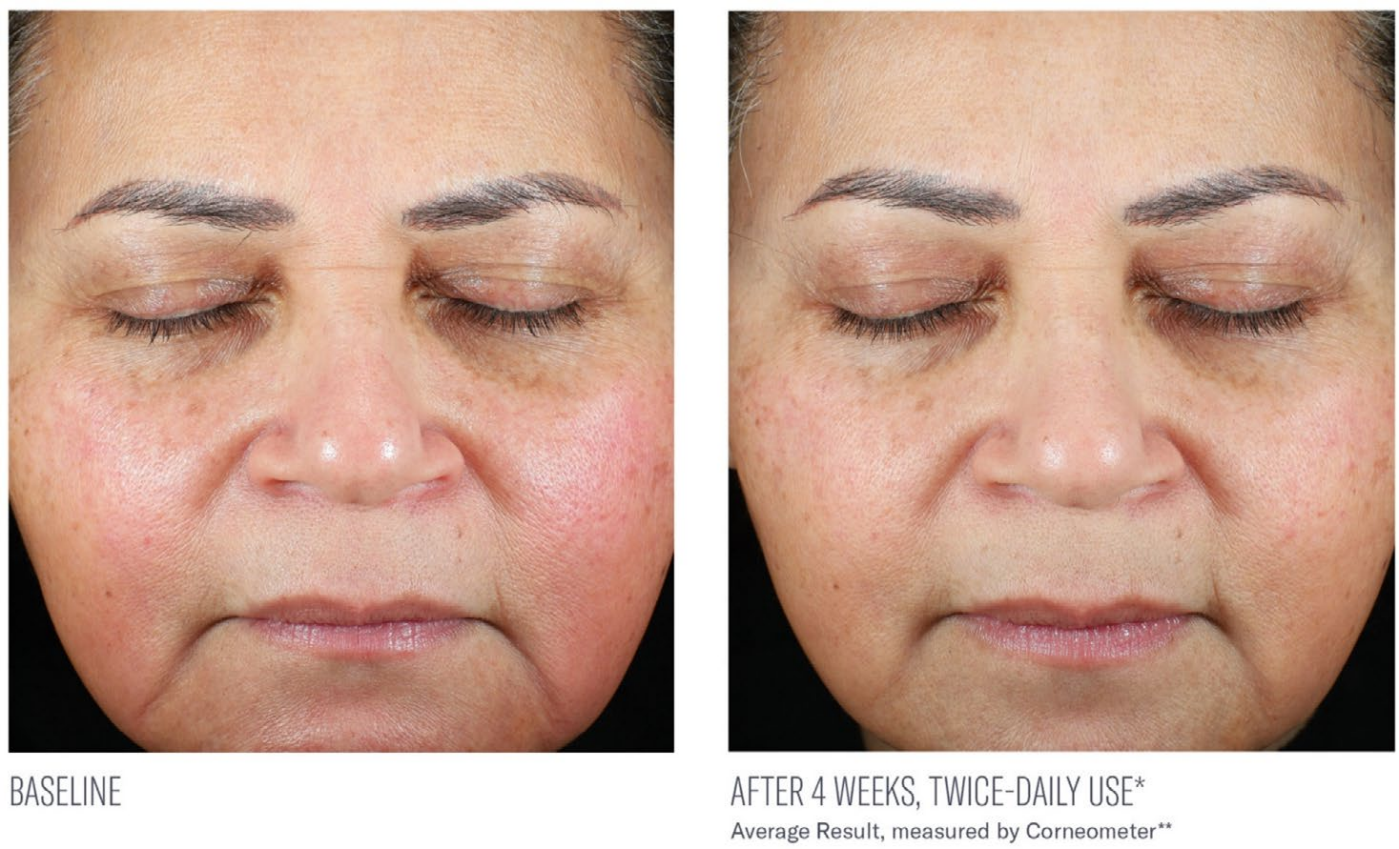
Visible improvements from baseline at 4 weeks. The average result is shown.

**FIGURE 4:**
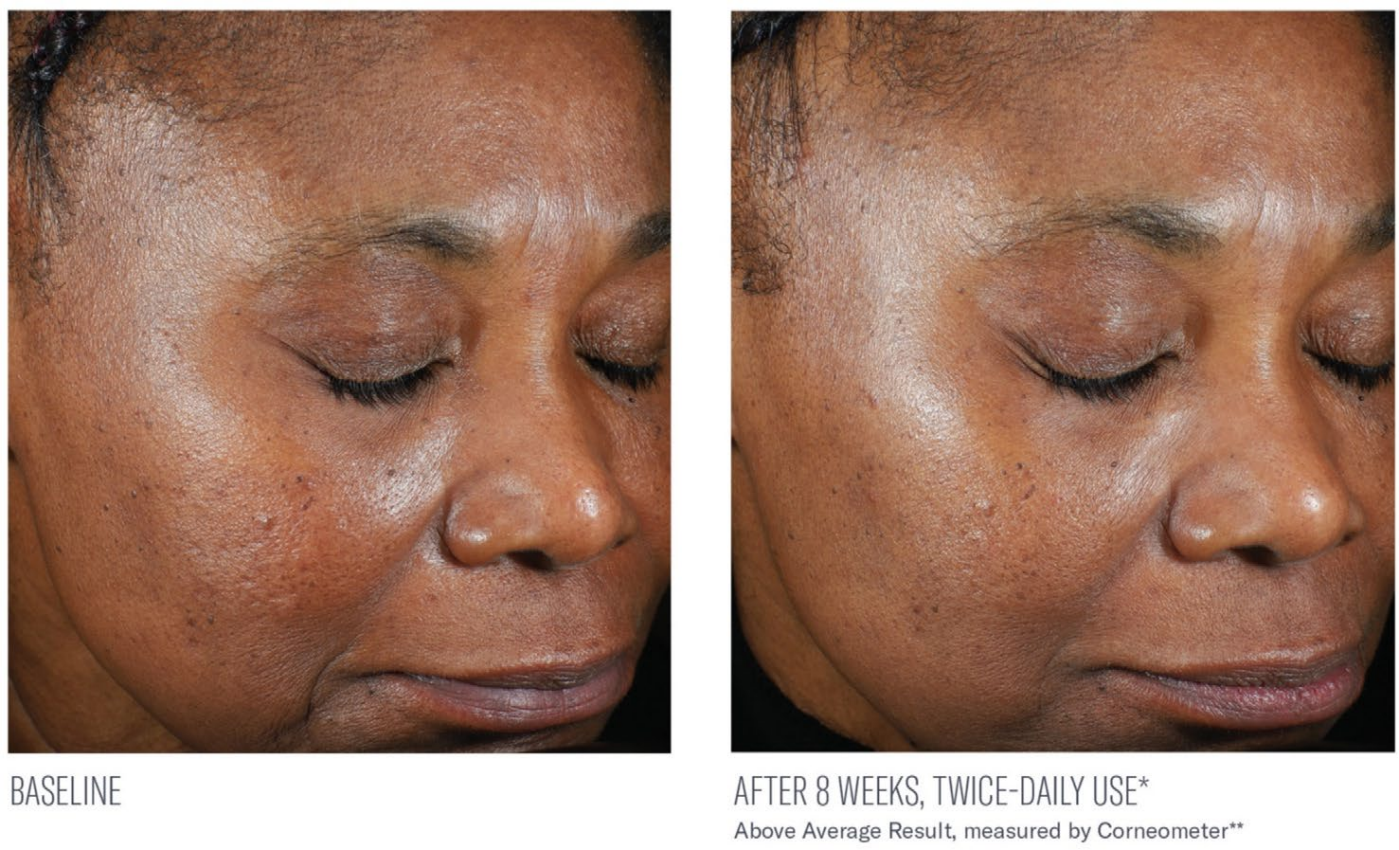
Visible improvements from baseline at 8 weeks. Above‐average result is shown.

**FIGURE 5:**
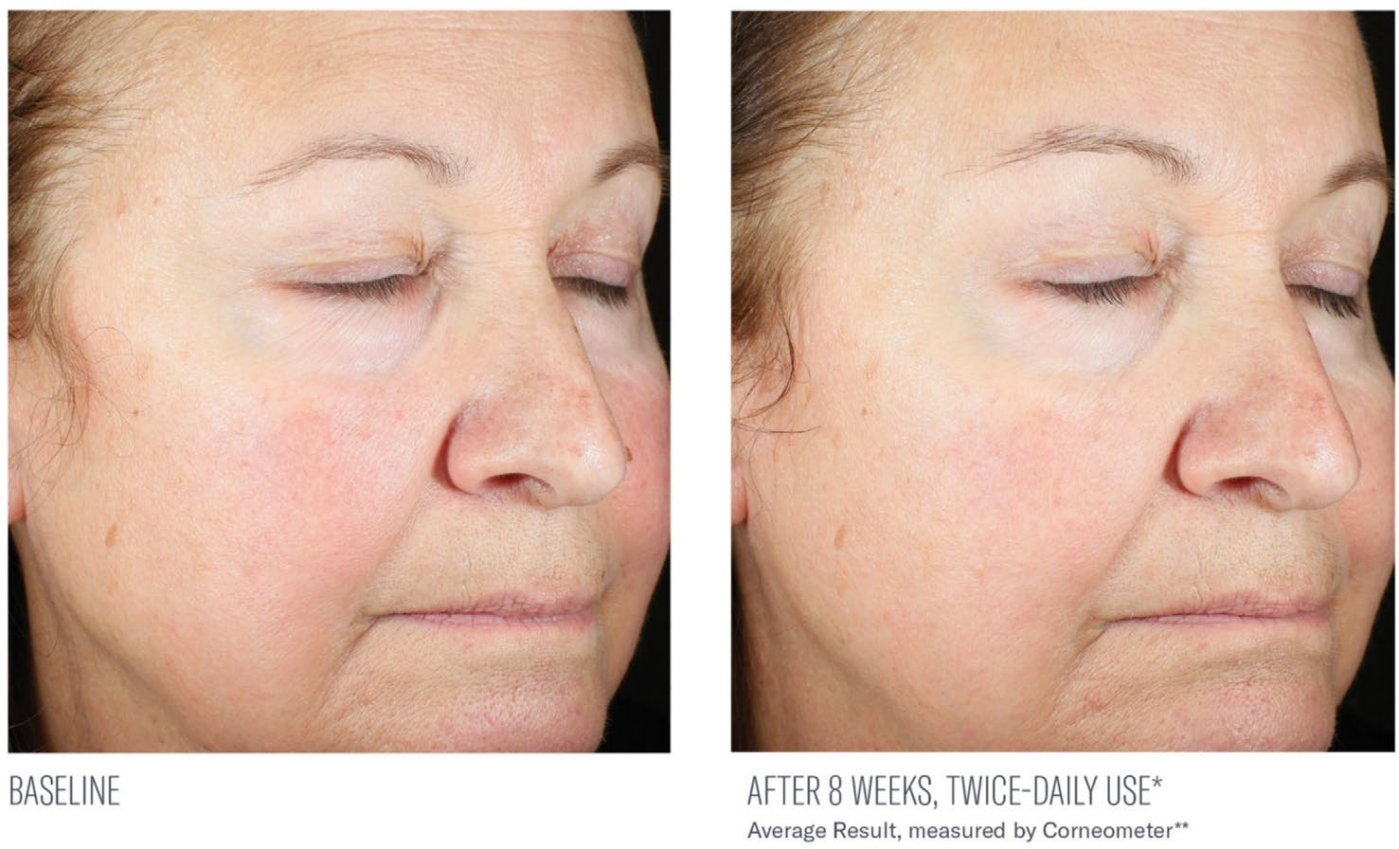
Visible improvements from baseline at 8 weeks. Average results are shown.

Significant mean percent improvements from baseline were demonstrated in skin hydration measurements at Weeks 2, 4, and 8 (41%, 38%, and 116%, respectively; all, *p* < 0.0001; Figure 2). Visible improvements in skin hydration are shown in subject images in Figures 3, 4, 5

#### 5.2.3 Subject Satisfaction

Thirty minutes following the initial application of the study product, 100% of subjects reported the study product absorbed nicely and had a pleasant texture/feel; 96% of subjects reported that their skin felt softer, 89% reported that their skin looked replenished, less dry, and healthier; and 81% reported their skin looked luminous and the texture looked smoother.

**FIGURE 6:**
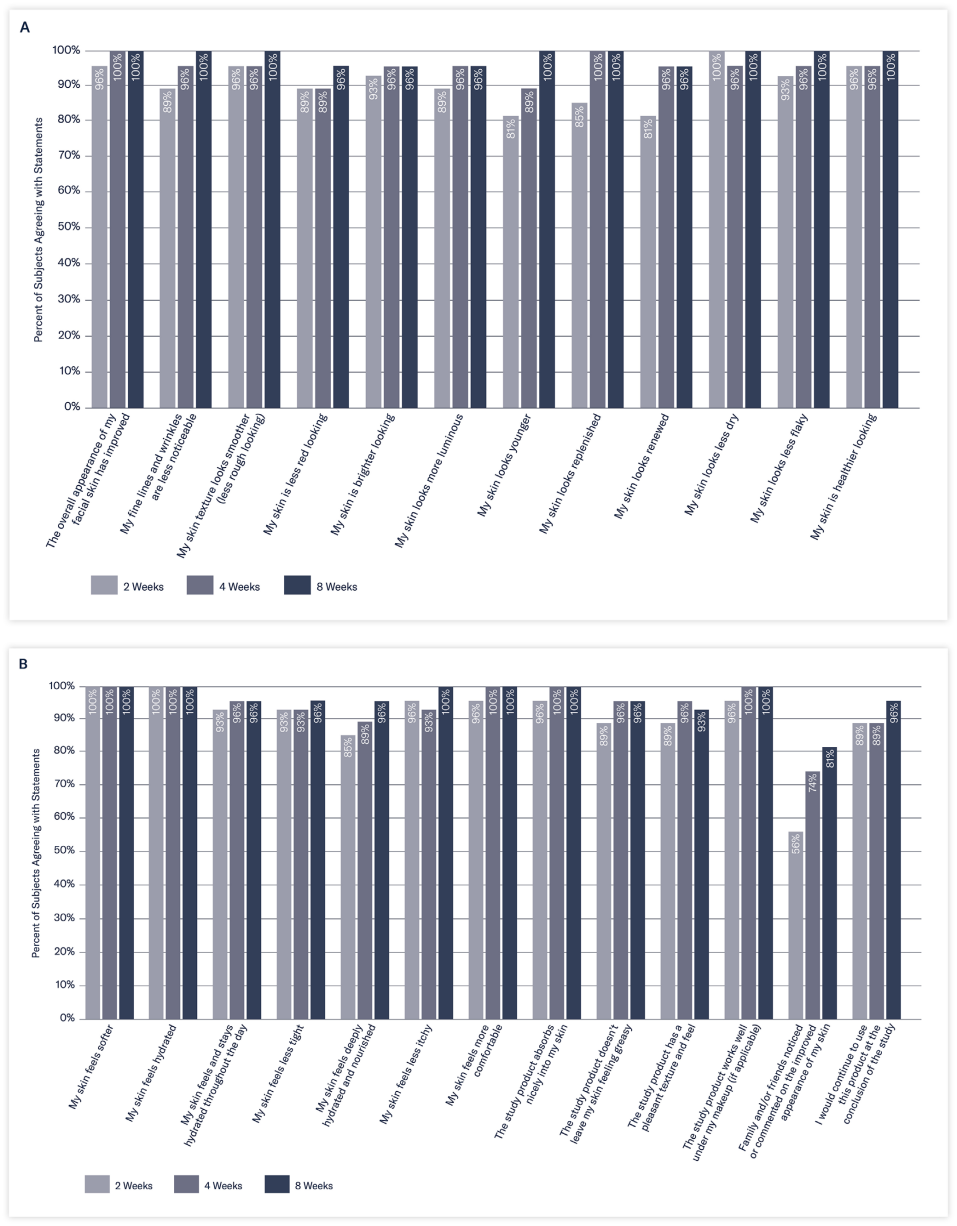
A and B Subject satisfaction over 8 weeks.

After 2 weeks of twice‐daily use, 100% of subjects reported their skin looked less dry and felt softer and hydrated, and 96% of subjects reported that the overall appearance of their skin improved, looked healthier, with a smoother‐looking texture (Figure 6A,B). In addition, 93% of subjects agreed that their skin felt and stayed hydrated throughout the day, and 89% reported their skin looked less red and more luminous.

After 8 weeks of twice‐daily use of the study moisturizer, 100% of subjects reported that their skin looked younger and healthier, with smoother texture and less noticeable fine lines/wrinkles, and 96% reported their skin felt deeply hydrated and nourished, noting that they would continue to use this product. Specifically, by the end of the study, 100% of subjects agreed the study product absorbed nicely into their skin and worked well under their makeup; 93% agreed it had a pleasant texture and feel; and 96% felt it was not greasy.

#### 5.2.4 Tolerability

Five subjects reported mild, transient tingling, stinging, or burning, with the majority of subjects reporting occurrence with the first application following a 3‐day washout period of their moisturizer per protocol. Mild, transient tightness (*n* = 1), itching (*n* = 1), and breakouts (*n* = 2) were also reported. No subject discontinued use of the study product or the study due to reported events and all events were resolved without sequelae.

## 6 Discussion

Skin requires sufficient hydration to maintain a healthy skin barrier and protect against the effects of environmental exposures and aging. The natural process of aging, coupled with chronic and cumulative exposure to environmental insults, contributes to a compromised skin barrier. Additional supplemental support is required to optimize and maintain a healthy skin barrier. As such, moisturizers are an essential component of an optimized skincare regimen necessary to facilitate and maintain sufficient water content. Effective moisturizers contain three critical elements that work together to moisturize the epidermis and underlying skin structures, specifically maintaining NMF, HA, and the natural lipid bilayer of skin to support skin hydration.

This moisturizing cream was developed utilizing foundational technology comprised of three key components to support natural skin moisturization. Ingredients employed in this technology supporting natural NMFs include a urea complex [24], a hygroscopic emollient, which helps enhance hydration and the integrity of the stratum corneum, sodium PCA and lactate, humectants that help hydrate and moisturize skin, and *Salicornia herbacea* extract, which supports hydration via stimulation of aquaporins [25]. Components of the technology that support the skin's lipid bilayer and barrier function include cholesterol, ceramides 1 and 2, the essential fatty acids (EFAs) linoleic and linolenic acids, carnitine, a naturally occurring amino acid, and squalene, which supports hydration and moisture retention [26]. Another humectant, sodium hyaluronate, is also an important element. Aminopropyl ascorbyl phosphate, a stable, water‐soluble form of vitamin C, is also incorporated based on its beneficial antioxidant properties and ability to help protect against HA degradation. Additional ingredients underpinning the technology to support skin hydration include glycerin, caffeine, and jojoba esters [24].

Previously reported in vitro and in vivo studies performed on the initial formulation that first employed this technology (TRMT) highlighted its mechanistic and hydrating capabilities and clinical effects in subjects with photodamaged skin [27]. Specifically, in vitro skin tissues pretreated with the original formulation demonstrated greater hydrating benefits via bioinstrumentation vs. tissues pretreated with a market‐leading moisturizer and demonstrated greater expression of key biomarkers associated with skin hydration and barrier support. Skin tissues pretreated with TRMT demonstrated greater expression of CLD, AQP, and HAS along with substantially lower expression of HYAL. Together, these findings demonstrated the activity of the product on key biomarkers related to skin barrier support and hydration. These benefits translated into visible clinical effects—including improvements to fine lines/wrinkles, erythema, skin brightness, and skin texture [27].

The moisturizer formulation described in the current study presented here (TRMT‐L) is an emollient‐enhanced moisturizing cream built on existing technology [27] that supports NMF, HA, and lipids with additional ingredients including glycine, niacinamide, and *Olea europaea* (olive) fruit extract to meet the needs of patients with moderately to severely dry, dehydrated skin who prefer a more emollient‐rich formulation. Both formulations utilizing this technology are formulated without fragrance masking agents or known ingredients that could potentially be irritating to a compromised barrier owing to age, environmental exposures, or use of exfoliating or other dermal irritants.

Building upon previous studies conducted, the aim of the in vitro and in vivo studies discussed herein was to confirm mechanistic targets via evaluation of key biomarkers, correlate these findings with objective clinical measures of hydration, and assess subject satisfaction. The gene expression analysis validated and confirmed enhanced expression of key biomarkers associated with support of the skin barrier, hydration, and protection from HA‐degrading enzymes. When clinically tested in subjects with moderate‐to‐severe dry, dehydrated skin over 8 weeks, twice‐daily use of the study moisturizer led to significant improvements from baseline in objective measurements of skin hydration and high levels of subject satisfaction.

## Disclosure

Dr. Draelos was the investigator in this study; DMs. Nelson is an employee of skinbetter science.

## Data Availability

The data that support the findings of this study are available on request from the corresponding author. The data are not publicly available due to privacy or ethical restrictions.
